# Two-Stream Modality-Based Deep Learning Approach for Enhanced Two-Person Human Interaction Recognition in Videos

**DOI:** 10.3390/s24217077

**Published:** 2024-11-03

**Authors:** Hemel Sharker Akash, Md Abdur Rahim, Abu Saleh Musa Miah, Hyoun-Sup Lee, Si-Woong Jang, Jungpil Shin

**Affiliations:** 1 Department of Computer Science and Engineering, Pabna University of Science and Technology, Rajapur 6600, Bangladesh; hemelakash472@gmail.com (H.S.A.); rahim@pust.ac.bd (M.A.R.); 2 School of Computer Science and Engineering, The University of Aizu, Aizuwakamatsu, Fukushima 965-8580, Japan; 3 Department of Game Engineering, Dongeui University, Busanjin-Gu, Busan 47340, Republic of Korea; 4 Department of Computer Engineering, Dongeui University, Busan 47340, Republic of Korea

**Keywords:** keypoint mesh model, HIR, SRGB-Model, MobileNetv2, deep bidirectional LSTM

## Abstract

Human interaction recognition (HIR) between two people in videos is a critical field in computer vision and pattern recognition, aimed at identifying and understanding human interaction and actions for applications such as healthcare, surveillance, and human–computer interaction. Despite its significance, video-based HIR faces challenges in achieving satisfactory performance due to the complexity of human actions, variations in motion, different viewpoints, and environmental factors. In the study, we proposed a two-stream deep learning-based HIR system to address these challenges and improve the accuracy and reliability of HIR systems. In the process, two streams extract hierarchical features based on the skeleton and RGB information, respectively. In the first stream, we utilised YOLOv8-Pose for human pose extraction, then extracted features with three stacked LSM modules and enhanced them with a dense layer that is considered the final feature of the first stream. In the second stream, we utilised SAM on the input videos, and after filtering the Segment Anything Model (SAM) feature, we employed integrated LSTM and GRU to extract the long-range dependency feature and then enhanced them with a dense layer that was considered the final feature for the second stream module. Here, SAM was utilised for segmented mesh generation, and ImageNet was used for feature extraction from images or meshes, focusing on extracting relevant features from sequential image data. Moreover, we newly created a custom filter function to enhance computational efficiency and eliminate irrelevant keypoints and mesh components from the dataset. We concatenated the two stream features and produced the final feature that fed into the classification module. The extensive experiment with the two benchmark datasets of the proposed model achieved 96.56% and 96.16% accuracy, respectively. The high-performance accuracy of the proposed model proved its superiority.

## 1. Introduction

Human interaction recognition (HIR) enables machines to observe, assess, and categorise human activities using advanced computational techniques. These methods allow for the real-time understanding of human behaviours, making HIR crucial for applications like video surveillance, abnormal behaviour detection, action-based video retrieval, and healthcare monitoring [1,2,3,4]. A key challenge in HIR is feature extraction, as system performance heavily depends on the quality of extracted features. While jumping for a football header may resemble dodging ropes, subtle differences in body movements can be identified by analysing sequences of frames [1,5]. HIR approaches include video-based, sensor-based, and hybrid models [6,7]. Video-based methods, often using RGB cameras and CNNs, struggle with issues like occlusion, lighting variability, and capturing complex multi-person interactions [5,8]. Sensor-based methods can track specific movements but require users to wear devices, limiting their scalability for real-time applications. They also lack the contextual information video data provides, reducing accuracy in cluttered environments [7]. Video-based systems, particularly those using surveillance cameras, excel at tracking body movements and capturing the dynamics of human activity. Unlike sensors or still images, video analysis allows for a more complete understanding of actions by tracking movements over time [8,9]. Many researchers have recently used machine learning tools with diverse hand-crafted features to develop an effect system. Yun et al. (2012) extracted joint, plane, and velocity features. Then, they applied support vector machine (SVM) classifiers [10]. Finally, they used the Two Person Interaction Kinect Dataset to evaluate their model. The joint feature achieved an accuracy of 80.03%, while plane and velocity features alone reached 73.80% and 48.03%, respectively. When combining different features like joint and plane or joint and velocity, the results slightly varied, with the highest being 80.03%. However, their performance accuracy is not as high as people expected. To improve the performance accuracy, Hu et al. extracted a combination of joint, plane, and velocity features, attaining an accuracy of 81.67% with SVM and 83.33% using a multiple instance learning (MIL) approach using Two Person Interaction Kinect datasets [11]. To improve the performance accuracy, Saha et al. used rotation-invariant and rotation-variant phenomena as features with an SVM classifier, resulting in an accuracy of 90.00% [12]. Manual feature extraction faces challenges such as rigid design, limited ability to capture high-level details in visual data, and difficulty in performing both extraction and classification effectively [13]. Traditional machine learning approaches for HIR often rely on hand-crafted features, which are limited in capturing the complex spatial and temporal relationships between body parts. These systems struggle to generalise across diverse environments, as hand-crafted features lack flexibility and fail to adapt to variations in human actions. Additionally, they often require extensive manual effort for feature extraction, which makes the process time-consuming and less efficient. Deep learning technology has recently demonstrated significant improvements in HIR by automating feature extraction and eliminating the limitations of hand-crafted features. Deep learning models, particularly CNNs and LSTMs, have been widely used in RGB video-based HIR systems to enhance the accuracy of action recognition by analysing patterns of body movements over time [2,3,14,15]. These models have effectively captured temporal dynamics, showing notable improvements in performance. For instance, Li et al. (2020) used torso, hand, arm, and leg movements as features, achieving 90.4% accuracy with an LSTM classifier and 90.6% with an LSTM-CRF classifier [16]. However, video-based methods often suffer in unstable environments with noisy data, where the visual quality degrades or objects occlude parts of the human body. To address these limitations, researchers have shifted toward skeleton-based HIR systems, which use body joint positions instead of pixel-level data. These systems, leveraging models like CNNs, LSTMs, and transfer learning, have shown promise in improving the robustness of human activity recognition [10,11,12,17,18]. Skeleton-based approaches excel at capturing key body movements but still face challenges in recognising subtle interactions and dynamic real-world scenarios, particularly in the presence of complex spatial and temporal relationships between different body parts, such as hands, legs, and head movements. Despite progress in HIR using either RGB or skeleton-based methods, challenges remain, particularly in achieving high accuracy due to the complex spatial and temporal relationships between body parts, such as the hands and legs. RGB-based methods capture visual context well but struggle with occlusion and noisy data, while skeleton-based systems provide a simplified representation but lack contextual visual information. Both approaches often fail to capture the complete dynamics of human interactions. Additionally, integrating multiple modalities has been complex, leading to suboptimal performance in multimodal fusion [19,20]. These challenges highlight the need for robust, adaptable models capable of handling dynamic real-world conditions. Moreover, HIR is complicated by the difficulty of capturing a person’s mobility and mental state, and current systems lack effective feature extraction methods. While multimodal fusion—combining RGB, skeleton, and motion data—has been successfully applied in other domains [18,21,22,23], no prior work has effectively integrated RGB and skeleton features to enhance HIR.

To address these challenges, we propose a two-stream deep learning-based HIR system that combines both RGB and skeleton features. Our approach leverages the complementary strengths of joint skeleton-based hierarchical features and pixel-based hierarchical features to improve accuracy and robustness. This novel fusion strategy represents a significant advancement in HIR, contributing to the state of the art by enhancing both performance and reliability.

Our main contribution is below:

1 We propose a novel two-stream deep learning-based human interaction recognition (HIR) system that integrates RGB and skeleton-based hierarchical features to address the challenges of video-based HIR, such as action complexity, motion variations, different viewpoints, and the lack of effective feature extraction methods.
2 Our system utilises two distinct streams: the first stream employs YOLOv8-Pose for human pose extraction, enhanced by stacked LSM modules and a dense layer. The second stream utilises the Segment Anything Model (SAM) for segmented mesh generation, followed by an integrated LSTM-GRU network for long-range dependency feature extraction. The first stream processes images extracted from videos using the YOLOv8-Pose pre-trained model, which generates bounding boxes around humans and identifies key body points. Subsequently, it evaluates proximity and collisions between these bounding boxes, particularly focusing on instances where individuals or objects come into close contact or collide. This standardised procedure lays the foundation for the initial phase of our model. Our approach combines YOLOv8 Pose for precise human keypoint detection and bounding box creation with SAM for image segmentation, refining spatial relationships through a custom filter for meshes and keypoints. The filtered mesh is then processed by an ImageNet model to produce a comprehensive feature vector.
3 We introduce a novel custom filter function to enhance computational efficiency by eliminating irrelevant keypoints and mesh components, thereby improving the overall performance of the HIR system.
4 Through extensive experimentation, our proposed model demonstrates superior performance on the TPIK and HAR Video datasets, achieving 96.56% and 96.16% accuracy, respectively, thereby confirming its effectiveness and reliability in video-based HIR.

The remainder of this paper is organised as follows: Section 2 reviews recent advancements in human interaction recognition. Section 3 provides a detailed description of the dataset. Section 4 elaborates on the proposed methodology, covering data preprocessing, various techniques, positional activity and skeleton extraction, segmentation, and classification methods. Section 5 presents the experimental evaluation, including performance results and accuracy. Finally, Section 6 concludes the paper.

## 2. Related Work

Human interaction recognition (HIR) has evolved significantly, with researchers exploring various machine learning and deep learning approaches [21,24,25,26,27,28,29,30]. Traditional machine learning methods used in HIR typically relied on hand-crafted features. For instance, Yun et al. [10] extracted joint, plane, and velocity features and applied support vector machine (SVM) classifiers. Although this approach achieved moderate accuracy (80.03% for joint features), the system struggled to perform well in dynamic environments. Similarly, Hu et al. [11] combined joint, plane, and velocity features, reaching 83.33% accuracy with a multiple instance learning (MIL) approach. However, the performance gains were marginal, and the models remained inflexible, highlighting the limitations of hand-crafted feature extraction in real-world scenarios. Saha et al. [12] improved accuracy to 90.00% by incorporating rotation-invariant and rotation-variant features, but the manual feature extraction process still proved time-consuming and lacked adaptability. These traditional methods require extensive manual effort and struggle with complex spatial and temporal relationships between body parts, making them unsuitable for large-scale or real-time applications. The mentioned methods relied heavily on hand-crafted features, but these approaches often struggled to generalise across diverse environments and failed to capture complex human motions effectively. Recently, deep learning has emerged as a powerful tool for automating feature extraction, significantly improving the accuracy and efficiency of HIR systems. Deep learning has addressed many of these challenges by automating feature extraction. CNNs and LSTMs have been widely adopted in RGB video-based HIR systems to capture body movements over time, significantly improving performance. Li et al. [16] utilised torso, hand, arm, and leg movements, achieving 90.4% accuracy with an LSTM classifier and 90.6% with an LSTM-CRF classifier. However, RGB-based methods still suffer in unstable environments, particularly when data are noisy, occluded, or inconsistent, limiting their application in real-world settings. To address these limitations, researchers have turned to skeleton-based HIR systems, which use body joint positions rather than pixel-level data. Skeleton-based systems excel in capturing key body movements and have shown promise in improving the robustness of human activity recognition [10,11,12,17,18]. Despite this progress, skeleton-based systems alone often lack the rich contextual information provided by RGB data, making it difficult to recognise subtle interactions or handle complex real-world scenarios involving multiple agents or varying environments. Even with advancements in both RGB and skeleton-based methods, several challenges persist. RGB-based systems capture visual context but struggle with occlusion and noisy data, while skeleton-based methods simplify the representation of human actions but miss important contextual information, such as background or environmental interactions. Moreover, integrating multiple modalities has proven to be complex, leading to suboptimal performance in multimodal fusion [19,20]. This creates a gap in the existing literature, where no approach has successfully integrated both RGB and skeleton features in a unified system for HIR, particularly in real-time applications. We propose a two-stream deep learning-based human interaction recognition (HIR) system that combines RGB and skeleton-based features, addressing the limitations of existing approaches. The first stream uses YOLOv8-Pose for precise human keypoint detection and pose extraction, while the second stream leverages the Segment Anything Model (SAM) for segmented mesh generation. This fusion enables better spatial and temporal representation of human actions, enhancing both accuracy and robustness.

- Stream 1 (YOLOv8-Pose): detects key body points, generating bounding boxes and evaluating interactions between individuals or objects.
- Stream 2 (SAM-based segmentation): creates meshes processed by an LSTM-GRU network to capture long-range dependencies, accounting for both motion and environmental context.

We also introduce a custom filter function to eliminate irrelevant keypoints and mesh components, further boosting computational efficiency. The advantages of our proposed method, including the custom filtering and two-stream deep learning model, are given below:

- Multimodal fusion: by integrating RGB and skeleton data, our system provides a more robust representation of human interactions, overcoming the limitations of single-modal approaches.
- Improved accuracy and efficiency: the combination of YOLOv8-Pose and SAM enhances feature extraction, achieving 96.56% accuracy on a benchmark dataset.
- Custom filtering: our custom filter improves real-time performance by removing irrelevant data and optimising computational efficiency.

Our two-stream HIR system offers a significant improvement over previous methods by effectively combining RGB and skeleton-based features. This approach enhances accuracy, robustness, and efficiency, contributing to state-of-the-art advancements in human interaction recognition.

## 3. Dataset

We used two datasets for evaluating the proposed model: the Two Person Interaction Kinect Dataset and the Kaggle Human Activity Dataset, which are given below.

### 3.1. Two Person Interaction Kinect (TPIk) Dataset

**Figure 1:**
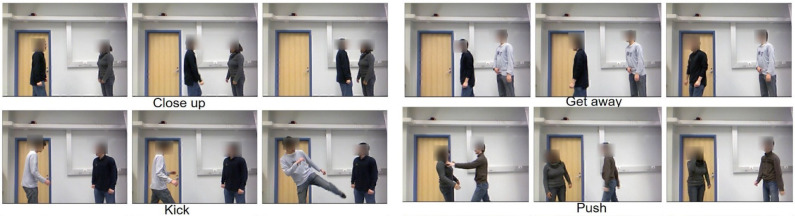
Example of the action-based interaction of the datasets [31].

Human interaction recognition is revolutionising surveillance, human–computer interaction, and video retrieval applications. Depth sensors, such as the Microsoft Kinect, perform real-time full-body tracking for effective activity detection that researchers use for various data collections. In this study, the “Two Person Interaction Kinect Dataset” (https://www.kaggle.com/datasets/dasmehdixtr/two-person-interaction-kinect-dataset (accessed on 31 October 2024) was employed to evaluate features commonly used for indexing and retrieving motion capture data. This dataset allowed us to assess the effectiveness of our proposed techniques in the real-time detection of interaction activities, focusing on key motion features and their applicability in human interaction recognition tasks. This dataset comprises eight distinct actions: close up, get away, kick, push, shake hands, hug, give a notebook, and punch. These actions are extracted from video sources and provide a realistic and dynamic training foundation. Each action is carefully selected to cover various human movements, ensuring comprehensive model training and classification capabilities for real-world applications. Figure 1 shows the sample of this dataset.

### 3.2. HAR Video Dataset

For evaluating the proposed model, we utilised a publicly available human activity recognition (HAR) video dataset, sourced from Kaggle (available at https://www.kaggle.com/datasets/sharjeelmazhar/human-activity-recognition-video-dataset (accessed on 31 October 2024). This dataset, created by a third party, is being reused for our research purposes. It consists of 1113 video clips representing seven distinct human activities, including clapping, meeting and splitting, sitting, standing still, walking, walking while reading a book, and walking while using a phone. Each video is labelled with its corresponding activity class, making it ideal for supervised learning tasks. The dataset is organised into a root directory containing videos for each of these seven classes, capturing a range of human actions, ranging from simple activities like sitting or standing still to more complex behaviours such as walking while reading a book or using a phone. The primary objective of this dataset is to support research in activity recognition, allowing machine learning models to accurately classify human activities. Given the growing importance of computer vision and deep learning techniques, this dataset is essential for improving model accuracy and robustness by providing diverse and comprehensive activity data.

## 4. Proposed Method

**Figure 2:**
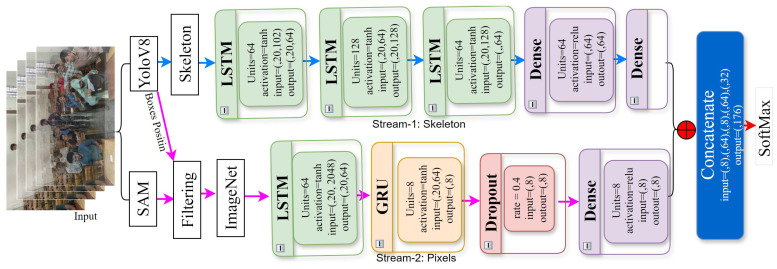
Proposed of the working flow architecture.

In this research, we propose a novel two-stream deep learning-based human interaction recognition (HIR) system that integrates RGB and skeleton-based hierarchical features to address key challenges in video-based HIR, such as action complexity, motion variations, diverse viewpoints, and ineffective feature extraction methods. Figure 2 shows the workflow architecture of the proposed model. The system is structured as follows:

- **RGB stream pose extraction:** The first stream employs the YOLOv8-Pose model for human pose extraction. This module processes video frames and generates bounding boxes around detected humans, identifying key body points such as joints and limbs. These keypoints are used to assess proximity and collisions between individuals or objects, especially in scenarios involving close contact or interaction. To refine these pose features further, stacked long short-term memory (LSTM) modules and a dense layer are applied, capturing complex spatial–temporal relationships for the accurate recognition of human poses and interactions.
- **Skeleton stream segmentation and mesh generation:** The second stream uses the Segment Anything Model (SAM) to generate segmented meshes from input images. These segmented outputs are passed through an integrated LSTM-GRU network, which captures long-range dependencies and dynamic temporal patterns in human movements. This stream focuses on the structural relationships between body parts across frames.
- **Custom filter function:** A custom filter function is applied to both streams to enhance computational efficiency. This filter eliminates irrelevant keypoints and mesh components, reducing the amount of data processed while maintaining accuracy. The filtered mesh data are processed through an ImageNet model, producing a comprehensive feature vector that captures both spatial and temporal aspects of human activities.
- **Integrate RGB and skeleton features:** The outputs from the two streams (RGB and skeleton) are integrated to form a unified feature representation [32]. The RGB stream provides crucial visual context, such as object appearance and surrounding environment, while the skeleton stream focuses on the structural and dynamic movements of the body. By combining these two streams, the fused representation captures both the spatial relationships from the RGB data and the temporal dynamics from the skeleton features. This unified feature vector is then passed to a final classification module, where the system accurately recognises human activities. This multimodal fusion enables the system to overcome challenges posed by occlusion, noisy environments, and complex multi-agent interactions, significantly enhancing recognition accuracy and robustness in diverse real-world scenarios.
- **Optimisation for efficiency:** To further optimise system performance, the custom filter function plays a crucial role in reducing computational overhead. By eliminating irrelevant features, the system processes data more efficiently, ensuring real-time performance while maintaining high accuracy.

We described each stream and integrated RGB and skeleton features with the optimisation listed in detail in the section below.

### 4.1. Preprocessing: Augmentation

We converted each video into approximately 30 frames, resulting in 280 curated samples, each consisting of 20 frames, to capture the fine details of the actions. This structure enhances the dataset’s capability for training and evaluation by enabling a deeper exploration of the temporal dynamics present in each action. To further enrich the dataset, we applied data augmentation, a technique widely used in machine learning and computer vision to increase the diversity of a training dataset through various transformations while preserving the underlying label or meaning. Standard augmentation techniques include rotation, flipping, zooming, translation, brightness and contrast adjustments, noise addition, and colour jittering. These transformations create a more diverse set of training examples, helping models learn invariant features and improving robustness to real-world variations. This approach is particularly beneficial when labelled data are limited as it effectively enlarges the dataset without requiring additional labelled samples. Table 1 outlines the augmentation methods and their respective ranges.

Augmentation techniques, such as rotation (±30°, ±20°, ±10°), scaling (with factors of 1.1, 1.2, and 1.3 for expansion, and 0.9, 0.8, and 0.7 for compression), and horizontal flips, expanded the dataset to 3640 samples, enhancing its diversity and robustness. This enriched dataset strengthens the model’s ability to handle variations and provides a solid foundation for training models to adapt to real-world dynamics. The approach underscores our commitment to realism and adaptability in action recognition, fostering advancements in artificial intelligence for practical applications.

### 4.2. YOLov8

YOLOv8-Pose is an advanced model designed for estimating the locations and orientations of human body parts in an image, including the head, shoulders, elbows, wrists, hips, knees, and ankles. It builds upon YOLOv8, a widely recognised object detection model known for its speed and accuracy [33]. Unlike traditional methods that rely on predefined anchor boxes, YOLOv8-Pose employs an anchor-free Ultralytics head, predicting object centre points and sizes directly. The model splits the detection task into two branches: one for bounding boxes and another for keypoints. This dual-branch architecture effectively handles different levels of complexity for each task. Trained on the COCO keypoints dataset, YOLOv8-Pose provides annotations for 17 keypoints per person across various activities and visibility conditions, making it highly versatile for human pose estimation.

YOLOv8-Pose excels due to its foundation on YOLOv8, a model celebrated for its balance of speed and accuracy in object detection. By leveraging an anchor-free approach and splitting detection tasks into specialised branches, precision is enhanced in identifying and localising human body parts. The model’s training on the extensive COCO keypoints dataset ensures robustness and versatility in pose estimation across different activities and scenarios.

YOLOv8-Pose offers several distinct advantages over other pose estimation methods:

- **Speed and accuracy:** inherits YOLOv8’s renowned capabilities for fast and precise detection.
- **Anchor-free detection:** bypasses the limitations of predefined anchor boxes, facilitating more flexible and efficient object detection.
- **Dual-branch architecture:** separates bounding box and keypoint detection, effectively managing the complexities of each task.
- **Robust training data:** trained on the COCO keypoints dataset, which includes diverse annotations and visibility conditions, improving the model’s generalisation across various pose estimation tasks.

Overall, YOLOv8-Pose’s efficient architecture, robust training, and high performance make it a compelling choice for accurate and reliable human pose estimation.

#### Position of Activity and Skeleton Extraction

**Positioning using YOLOv8:** In our feature extraction pipeline, we leverage YOLOv8 to detect human positions with high precision. The process begins with identifying all human boundary boxes within the given dataset. This initial step ensures a comprehensive scan to locate potential human figures accurately. Subsequently, we filter and isolate the boundary boxes where individuals are actively engaged in dynamic actions. This discerning step acts as a critical filter, honing in on specific instances that encapsulate human movement and activity. By focusing on active individuals, we enhance the relevance of the extraction of subsequent key points.

**Skeleton extraction:** Once the refined subset of active human actions is established, we proceed to extract the relevant keypoints associated with these discerned boxes. This meticulous approach ensures that our keypoints model is applied exclusively to instances characterised by human action, enhancing the precision of the extracted features. In this intricate process, each frame encapsulates the dynamic interplay of two individuals, each uniquely characterised by 17 keypoints, denoted as (x, y, z). Consequently, every frame is a rich tapestry of 102 features (2 persons * 17 keypoints * 3 coordinates), providing a comprehensive representation of human movement. This strategic approach to skeleton extraction ensures high precision and relevance, focusing on dynamic human actions and delivering a robust dataset for further analysis [21,34,35].

### 4.3. Stream-1: Skeleton-Based Stream

In the first stream, skeletal keypoints were extracted using YOLOv8 and subsequently processed with LSTM networks [2,22,27]. This stream is distinguished by its use of MediaPipe for hand position estimation, which excels in real-time performance and captures intricate hand gestures with high precision. The sequence employed in this stream includes three LSTM modules and two dense layer modules to process the collected joint skeleton points. This combination effectively captures the nuances of sequential data and accurately represents the gestures. As a result, the total number of features per activity, considering each action and individual, amounts to approximately 307,200. Additionally, the third stream leverages MediaPipe for hand posture analysis, focusing on hand position and finger placement. This approach yielded 258 keypoints per image, resulting in a total of 38,700 features per activity.

### 4.4. Stream-2: Pixels-Based Stream

A key element of this stream’s architecture is the ImageNet module. ImageNet, with its extensive collection of annotated images, is crucial for extracting generalised and discriminative features that bolster the model’s robustness. These features are then fed into LSTM and GRU models, which adeptly handle both short-term and long-term dependencies. This process is further refined by a dense layer that introduces additional depth, allowing the network to capture and learn complex patterns.

### 4.5. SAM

The semantic annotation model (SAM) is a powerful foundation model designed for image segmentation tasks. Leveraging a vast dataset of 11 million images and over 1 billion masks, SAM excels in generating accurate and high-quality segmentation masks. Image segmentation involves dividing an image into segments, each comprising pixels that share common characteristics such as colour, intensity, texture, or shape. SAM operates through three primary modules: an image encoder, a prompt encoder, and a mask decoder. In our process, SAM is utilised to segment images from a human activity dataset. The image encoder processes the input image to extract relevant features, while the prompt encoder incorporates contextual information to guide the segmentation. The mask decoder then generates precise segmentation masks. This modular architecture allows SAM to efficiently distil complex visual information into meaningful components, facilitating the enhanced analysis of human activities in the dataset. SAM is particularly effective for our goal of segmenting human activity dataset images due to its impressive speed and accuracy. Its extensive training on a large and diverse dataset ensures robust performance across a wide range of images and segmentation tasks. By breaking down intricate images into simpler, well-defined segments, SAM enhances the ability to analyse and understand human activities. This makes it an invaluable tool for detailed and accurate segmentation in human activity recognition, supporting further analysis and interpretation of the dataset.

#### 4.5.1. Box Filter

In our research, we tackle the challenge of processing video frames with multiple individuals, acknowledging that not every person in a frame is actively engaged in an action. We introduce a novel approach employing a custom box filter method to optimise computational efficiency and conserve valuable resources.

**Custom box filter method:** Our method involves the use of a three-dimensional box filter to segment human activity images effectively. The goal of this filter is to multiply the segmented image with the output of YOLOv8, focusing on individuals actively engaged in actions. To determine if two rectangular boxes are colliding, we use the following conditions based on the coordinates of their corners. Box 1 and Box 2 are expressed as the below Equations (1) and (2).

(1) $$\mathrm{Box}1=\{\left(a_{1},b_{1},c_{1}\right),\left(a_{2},b_{2},c_{2}\right),\left(a_{3},b_{3},c_{3}\right),\left(a_{4},b_{4},c_{4}\right)\}$$

(2) $$\mathrm{Box}2=\{\left(q_{1},r_{1},s_{1}\right),\left(q_{2},r_{2},s_{2}\right),\left(q_{3},r_{3},s_{3}\right),\left(q_{4},r_{4},s_{4}\right)\}$$

Here, $\left(a_{i},b_{i},c_{i}\right)$ are the coordinates of the corners of Box 1, and $\left(q_{i},r_{i},s_{i}\right)$ are the coordinates of the corners of Box 2. Two boxes are considered to be colliding if there is no gap between them in any dimension. The mathematical conditions for collision can be expressed as follows:

- **Collision in x-axis**:
  $$\max\left(a_{1},a_{2},a_{3},a_{4}\right)\geq \min\left(q_{1},q_{2},q_{3},q_{4}\right)$$
  $$\min\left(a_{1},a_{2},a_{3},a_{4}\right)\leq \max\left(q_{1},q_{2},q_{3},q_{4}\right)$$
- **Collision in y-axis**:
  $$\max\left(b_{1},b_{2},b_{3},b_{4}\right)\geq \min\left(r_{1},r_{2},r_{3},r_{4}\right)$$
  $$\min\left(b_{1},b_{2},b_{3},b_{4}\right)\leq \max\left(r_{1},r_{2},r_{3},r_{4}\right)$$
- **Collision in z-axis**:
  $$\max\left(c_{1},c_{2},c_{3},c_{4}\right)\geq \min\left(s_{1},s_{2},s_{3}\right)$$
  $$\min\left(c_{1},c_{2},c_{3},c_{4}\right)\leq \max\left(s_{1},s_{2},s_{3},s_{4}\right)$$

If all three conditions are true, the boxes are colliding in 3D space. Our custom box filter method circumvents the need to extract features from every individual in the frame by focusing on the spatial relationships between them. Unlike traditional methods that rely solely on two-dimensional (a, b) coordinates from YOLO for object detection, we incorporate a third dimension (c) derived from keypoints. This additional dimension adds depth to the analysis, enabling us to differentiate between individuals at varying distances from the camera. The rationale behind incorporating three-dimensional boxes is the inadequacy of relying solely on two-dimensional coordinates. Assessing proximity based only on (a, b) coordinates can lead to inaccuracies, such as individuals positioned further behind erroneously appearing close to each other, potentially misidentifying interactions.

- **Efficient filtering**: By considering the depth information provided by keypoints, our method accurately discerns whether individuals are genuinely close or merely share similar two-dimensional coordinates.
- **Reduced computational complexity**: This innovative approach significantly reduces computational complexity, enhancing the model’s efficiency without compromising accuracy.
- **Enhanced precision**: Effectively filtering out individuals not in close proximity optimises the identification of potential interactions, contributing to the model’s overall speed and precision.

Our research introduces a three-dimensional box filtering technique that leverages spatial relationships to identify and retain individuals likely to be involved in actions. This method streamlines the processing of video frames and enhances the overall performance of our model.

#### 4.5.2. ImageNet

ImageNet is a comprehensive dataset extensively used for training and evaluating computer vision models. Models trained on the ImageNet dataset have demonstrated exceptional performance across a range of computer vision tasks, such as image classification, object detection, and image segmentation. ImageNet is recognised as a key benchmark in the field, providing a standard measure for evaluating the effectiveness of computer vision systems. Algorithms are often evaluated by comparing their performance on the ImageNet validation dataset. Additionally, the dataset has grown in both size and diversity, with recent versions incorporating a larger number of images and categories.

#### 4.5.3. Long Short-Term Memory (LSTM)

Our approach uses a sequence of three LSTM networks. Long short-term memory (LSTM) networks, a form of recurrent neural network (RNN), are adept at capturing long-term dependencies within sequential data. These networks are commonly applied in fields such as natural language processing, speech recognition, and time series analysis [36]. LSTMs incorporate three primary gates that manage the flow of information into and out of the memory cells:

**Forget gate:** This gate decides whether to keep or discard information from the previous cell state. It assigns a value ranging from 0 to 1 to each element of the cell state, where 0 indicates “discard” and 1 indicates “keep”, based on the current input and the prior cell state.

**Input gate:** This gate manages the integration of new information into the cell state. It generates a candidate cell state by combining the current input with the previous cell state, which is then added to the cell state. These processes allow LSTMs to handle and leverage long-term dependencies in sequential data effectively.

**Output gate:** The output gate of an LSTM network decides what information from the cell state will be included in the final output. It produces the output by using both the current input and the updated cell state. The efficacy of LSTMs in hand motion detection arises from their ability to retain and utilise context from previous frames, which informs the interpretation of subsequent frames. Typically, an LSTM layer is applied following a convolutional or embedding layer to handle structured data, thereby improving the model’s ability to discern complex patterns and enhance overall accuracy.

**Figure 3:**
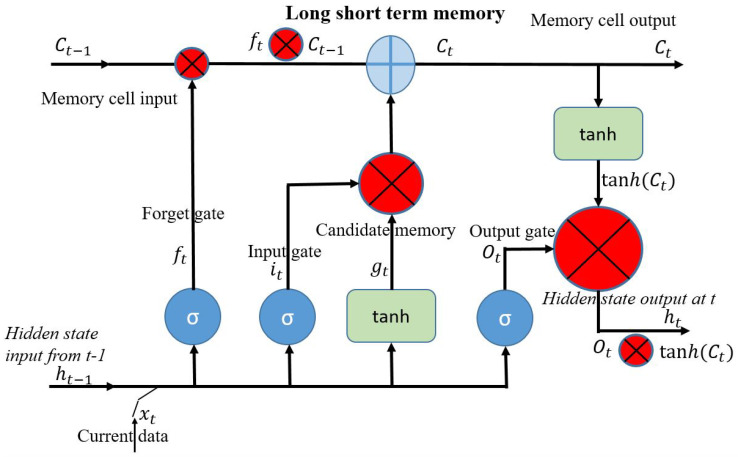
LSTM block diagram [3].

However, managing sequences of different lengths remains a challenge, often mitigated through the use of padding techniques. It is essential to balance model complexity with operational efficiency, particularly for real-time applications. LSTM networks exhibit considerable promise for high-performance sign language recognition because of their adeptness at processing sequential data, especially when combined with other neural network elements. Figure 3 shows the architecture of the LSTM module [2].

#### 4.5.4. Gated Recurrent Units (GRUs)

Gated recurrent units (GRUs) are a specialised type of recurrent neural network (RNN) designed to address the vanishing gradient problem that conventional RNNs encounter, which hinders their ability to learn long-term dependencies in sequences [37]. The GRU is optimised for computational efficiency through a simplified gating mechanism that retains essential information from previous time steps. The GRU architecture features two primary gates: the reset gate, which discards selected historical information, and the update gate, which controls the extent to which information from the previous state is retained. This design enables the effective capture of temporal patterns in sequential data. The behaviour of GRUs is governed by the equations detailed in Equations (1)–(4).

(3) $$Y_{t}=sigmoid\left(W_{y}\times \left[h_{t-1},x_{t}\right]\right)$$

(4) $$r_{t}=sigmoid\left(W_{t}\times \left[h_{t-1},x_{t}\right]\right)$$

(5) $$h_{t}=tanh\left(W_{h}\times \left[h_{t-1},x_{t}\right]\right)$$

(6) $$h_{t}=\left(1-y_{t}\right)\times h_{t-1}+Y_{t}\times h_{t}$$

where $x_{t}$ represents the input at time step *t*, and $W_{y}$, $W_{r}$, and $W_{h}$ are the weight matrices learned during the training process.

### 4.6. Final Feature

The goal was to synthesise the final features for the input into the dense classification layer by integrating the outputs from the three distinct streams. The resulting hybrid model represents a robust and efficient system for feature extraction, leveraging the unique strengths of each stream. This combined approach allows the model to handle the complex patterns present in the data effectively. The architecture of this innovative hybrid model is depicted in Figure 2 while the specific configuration of the GRU model is detailed in Table 2.

## 5. Experimental Evolution and Performances

In this section, we assess the proposed method using a widely recognised benchmark dataset for action recognition. Figure 1 provides a visual representation of sample actions from each dataset used in the evaluation.

### 5.1. Experimental Setup

Efficient model training depends significantly on hardware architecture. Deep learning models often use high-level libraries like TensorFlow [38,39] for handling computationally demanding tasks. In our study, we chose NVIDIA hardware for its strong support for deep learning computations, specifically using an NVIDIA GeForce RTX 3090 graphics card for evaluations. The proposed neural network architecture was developed in Python using the Keras and TensorFlow libraries. We evaluate the approach using accuracy and confusion matrix metrics, presenting results for each dataset and comparing them with state-of-the-art (STOA) methods.

### 5.2. Ablation Study

The ablation study is shown in Table 3, where we compared the performance of the proposed model using single-modality and multi-modality approaches for two datasets: the TPIk Dataset and the HAR Video Dataset. For the TPIk Dataset, the skeleton-only stream (Stream-1) achieved a validation accuracy of 71.00% with a validation loss of 1.82, while the RGB-only stream (Stream-2) yielded a higher accuracy of 95.00% and a validation loss of 0.14. However, combining both modalities (Skeleton+RGB) in a multi-stream configuration resulted in a higher accuracy of 96.56.00% with a loss of 0.1417. Moreover, for the HAR Video Dataset, the skeleton-only stream achieved 89.42% accuracy with a loss of 0.43, and the RGB-only stream attained 94.00% accuracy with a loss of 0.14. Notably, combining the skeleton and RGB modalities boosted the model’s performance, achieving a significantly higher accuracy of 96.16% with a loss of 0.10, demonstrating the effectiveness of multi-modality for this dataset.

### 5.3. Performance Accuracy with the TPIk Dataset

**Figure 4:**
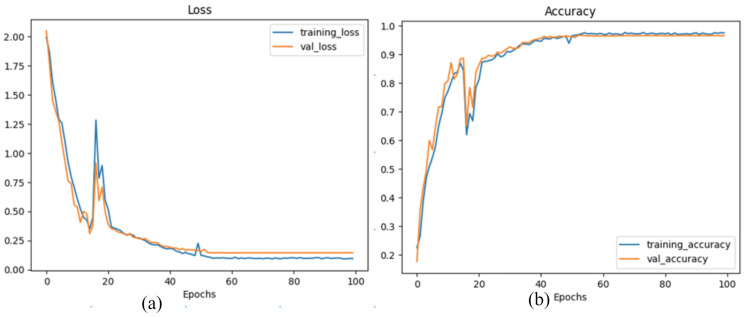
(**a**) Loss curve; (**b**) accuracy curve.

**Figure 5:**
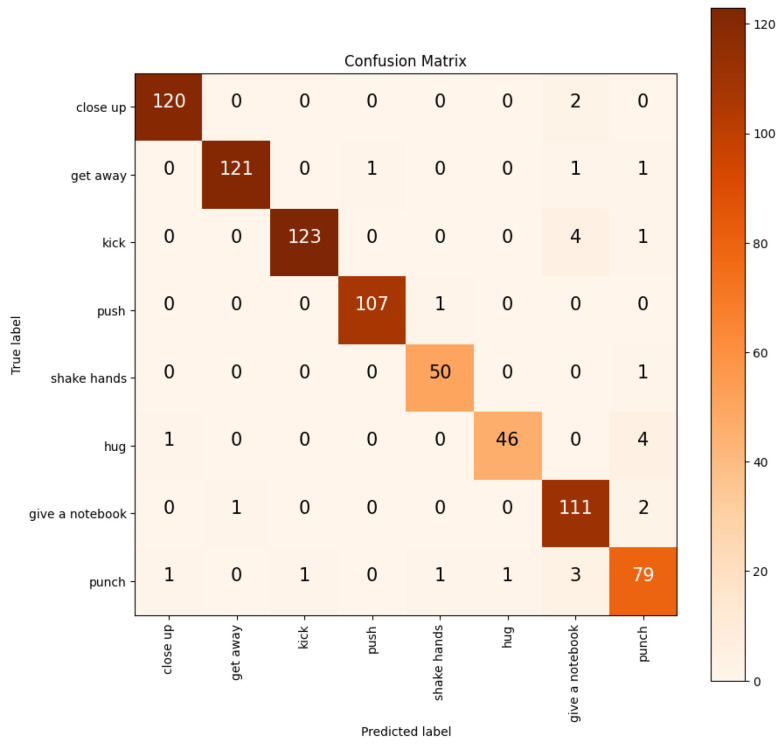
Confusion matrix of the proposed model.

This is an action detection model from video, so we fed 20 frames at a time to detect human activity. We trained the model for a total of 100 epochs. After 100 epochs, we obtained a learning accuracy of 97.51% and a prediction accuracy of 96.56%. For the loss function, we used the categorical cross-entropy function. In the training period, our model training loss was 0.0946, and after the training, its validation loss was 0.1447. Figure 4a,b illustrate the training and validation loss, as well as the accuracy curves, demonstrating the effectiveness and robustness of the proposed two-stream deep learning-based human interaction recognition (HIR) system. In addition, we also demonstrated the confusion matrix in Figure 5 to discern patterns of misclassifications within our model’s predictions. Notably, our model exhibited a tendency to misclassify actions, with the primary confusion being centred around the “Punch” action. Additionally, the “Hug” action was susceptible to mispredictions, often being confused with both “Punch” and “Give a Notebook” actions. This insight highlights areas for potential improvement in the model’s performance and underscores the need for targeted enhancements to accurately distinguish these specific actions.

In Table 4, we have a comprehensive overview of the accuracy for each action or activity predicted by our model, along with a count of false accusations. This provides valuable insights into the model’s performance across various actions and highlights instances where the model made inaccurate predictions. In Figure 4a, both the training and validation losses start at 2.00 in the first epoch and steadily decrease until epoch 18, reaching approximately 0.25. Although a temporary increase in loss occurs at epoch 20, with the training loss rising to 1.25 and validation loss rising to 0.99, both continue to decrease afterward, reaching approximately 0.10 by epoch 40. From epoch 60 onward, the training and validation losses stabilise at 0.10 and 0.12, respectively, maintaining these low values until epoch 100. This consistent decrease in loss and stabilisation over time underscores the model’s strong generalisation capability and its resistance to overfitting, even when dealing with complex human interactions. Figure 4b highlights the training and validation accuracy curves. Initially, in epoch 1, the training accuracy starts at 25%, while the validation accuracy is 15%. Both curves rise steadily, peaking at 89% by epoch 18. A slight drop to 62% occurs at epoch 20, but the model quickly recovers, with the accuracy returning to 89% by epoch 22. From this point, the accuracy continues to improve, reaching approximately 96% for both training and validation by epoch 100. The high performance of the proposed model can be attributed to several key innovations. The two-stream architecture efficiently extracts hierarchical features from both skeleton and RGB information, capturing complex interactions with remarkable accuracy. In the first stream, YOLOv8-Pose effectively handles human pose extraction, while the stacked LSTM modules and dense layers enhance feature representation. In the second stream, the combination of SAM, LSTM, and GRU modules captures long-range dependencies, refining the feature extraction process. Additionally, the custom filter function improves computational efficiency by removing irrelevant keypoints and mesh components, ensuring that the model processes only the most relevant data. The resulting accuracy of 96.56% on a benchmark dataset highlights the superiority of the proposed HIR system as it successfully overcomes the challenges of motion variation, different viewpoints, and environmental factors. The consistent improvement in both loss and accuracy, combined with the model’s stability across epochs, underscores its reliability and potential for real-world applications such as healthcare, surveillance, and human–computer interaction.

The confusion matrix in Figure 5 demonstrates the high performance and accuracy of the proposed human interaction recognition (HIR) model. For the “close up” activity, 120 trials were correctly classified, with only two being misclassified as “give a notebook”, showing the model’s strong ability to recognise subtle movements. Similarly, the “get away” activity shows 121 correct classifications, with just one being misclassified as “push”, further reinforcing the model’s precision. The “kick” activity presents 123 correctly classified trials, with five misclassifications—four into “give a notebook” and one into “punch”—which reflects the model’s capacity to handle complex and dynamic movements, even if some minor errors occur in similar activities. For the “push” activity, 107 trials were correctly classified, with just one trial being misclassified as “shake hands”, demonstrating the model’s accuracy in distinguishing between distinct physical interactions. The “shake hands” activity also performed well, with 50 correct classifications and only 1 being misclassified as “punch”. The “hug” activity showed 46 correct classifications and five misclassifications—one into “close up” and four into “punch”—which, although a slight challenge, is still indicative of the model’s capability in identifying human gestures. The “give a notebook” activity achieved 111 correct classifications, with five misclassifications—one into “get away” and two each into “punch” and “close up”. Lastly, the “punch” activity had 79 correctly classified trials, with seven misclassifications—one into each of “close up”, “kick”, “shake hands”, and “hug”; and three into “give a notebook”. These results emphasise the robustness and high performance of the proposed model as it consistently distinguishes between different activities with minimal misclassification. The few misclassifications that occur are mostly between similar or overlapping activities, a known challenge in human interaction recognition. However, the model’s overall precision and its ability to handle complex actions with high accuracy (96.56% on the benchmark dataset) underline the advantages of its two-stream architecture. By integrating both skeleton and RGB information and utilising custom filtering to remove irrelevant keypoints, the model proves to be a powerful and reliable tool for real-world applications such as healthcare, surveillance, and human–computer interaction. The minimal errors and high classification accuracy validate the proposed system’s superiority in understanding intricate human interactions.

#### State of the Art Comparison for TPIk Dataset

The state of the art comparison table presents various methodologies used for human interaction recognition (HIR) on the Two Person Interaction Kinect Dataset in Table 5. It highlights different approaches to feature extraction, classification methods, and their corresponding accuracy rates. Yun et al. (2012) experimented with multiple feature extraction techniques, such as joint, plane, and velocity features, using support vector machine (SVM) classifiers [10]. The joint feature achieved an accuracy of 80.03%, while plane and velocity features alone reached 73.80% and 48.03%, respectively. When combining different features like joint and plane or joint and velocity, the results slightly varied, with the highest being 80.03%. Hu et al. improved upon this by utilising a combination of joint, plane, and velocity features, attaining an accuracy of 81.67% with SVM and 83.33% using a multiple instance learning (MIL) approach [11]. Saha et al. further advanced the performance by applying rotation-invariant and rotation-variant phenomena as features with an SVM classifier, resulting in an accuracy of 90.00% [12]. Li et al. (2020) adopted a more detailed approach, utilising features such as torso, hand, arm, and leg movements, achieving 90.40% accuracy with an LSTM classifier and 90.60% with an LSTM-CRF classifier [16]. The proposed method in this study introduces a two-stream deep learning (DL) approach that combines RGB and skeleton-based features for human interaction recognition, resulting in a significantly higher accuracy of 96.56%. This demonstrates a substantial improvement over existing methods, showcasing the effectiveness of the deep learning model in capturing complex human interactions.

### 5.4. Performance Accuracy with HAR Video Dataset

We evaluated the proposed model using the human activity recognition (HAR) video dataset sourced from Kaggle, and Table 6 shows the performance accuracy for this dataset. The dataset contains seven activity labels, and all seven were used in our evaluation. The table below provides labelwise performance metrics, including precision, recall, F1 score, and accuracy. The model achieved a perfect performance of 100% for activities such as clapping, meeting and splitting, sitting, and standing still, showcasing its high accuracy in classifying these simpler actions. For the more complex activities such as walking, walking while reading a book, and walking while using a phone, the model achieved slightly lower accuracy rates. For instance, walking yielded an accuracy of 94.29%, with precision and recall values of 91.67% and 94.29%, respectively. Overall, the combined performance across all activities resulted in an impressive average accuracy of 96.16%, indicating the model’s robustness in recognising a diverse set of human activities within the dataset. This demonstrates the effectiveness of the proposed model for human activity classification tasks.

### 5.5. In-Depth Analysis and Discussion

The high overall accuracy (96.56%) achieved by the proposed model reflects the effectiveness of its two-stream architecture. The combination of YOLOv8-Pose for pose extraction and SAM for mesh segmentation allows the model to efficiently capture both spatial and temporal information, leading to precise recognition of complex human interactions. The stacked LSTM and GRU modules in both streams play a crucial role in capturing long-range dependencies, allowing the model to handle variations in motion and viewpoint effectively. However, as revealed by the confusion matrix, certain actions such as “Punch” and “Hug” were more prone to misclassification. For example, “Hug” was often confused with “Punch” and “Give a Notebook”, likely due to the overlapping physical gestures involved in these actions. This indicates that while the model excels at recognising most activities, it faces challenges when actions share similar body postures or movements. Such nuances highlight the need for future improvements, potentially through the incorporation of additional data sources or more sophisticated feature extraction techniques to better distinguish between similar actions. Additionally, while the model’s performance is generally robust, it performs slightly worse in recognising actions with subtle differences in movement or minimal contact, such as “Shake Hands”. This action had a few misclassifications, likely due to the limited range of motion compared with more distinct activities such as “Kick” or “Push”. Despite these minor shortcomings, the model’s ability to consistently classify a wide range of actions with high accuracy underscores its versatility and potential for real-world applications. The strength of this model lies in its ability to integrate RGB and skeleton-based features, allowing it to capture both detailed visual context and structural body movement information. This multimodal approach enables the model to handle complex scenarios with multiple actors or overlapping actions, which is a known limitation of many single-modal models. However, the model can face some difficulties in specific complex scenarios, particularly when distinguishing between actions that have very similar visual or motion characteristics. This is evident from the confusion between “Punch”, “Hug”, and “Give a Notebook”, where minor variations in movement were not always effectively captured. Future work could explore the integration of other modalities, such as depth information or contextual cues, to help disambiguate similar actions. Overall, the proposed HIR system demonstrates strong performance, with a high accuracy rate of 96.56%, effectively capturing complex human interactions in video data. The model’s consistent performance across a variety of actions, combined with its ability to generalise well, makes it a promising tool for real-world applications such as healthcare, surveillance, and human–computer interaction. However, the identified areas of misclassification suggest the potential for further improvement in distinguishing similar actions, providing a direction for future research.

## 6. Conclusions

In this study, we developed a novel two-stream deep learning-based human interaction recognition (HIR) system that integrates RGB and skeleton-based hierarchical features to address challenges in video-based HIR, such as action complexity, motion variations, and varying viewpoints. The proposed system uses two distinct streams, each capturing dynamic temporal patterns through hierarchical feature extraction. A custom-designed filter function eliminates irrelevant keypoints and mesh components, enhancing computational efficiency by focusing on the most relevant data. The filtered mesh data are processed using an ImageNet model, producing a feature vector that encapsulates both spatial and temporal aspects of human activities. The outputs from both streams are unified into a single feature representation, which is fed into the final classification module. By combining the strengths of RGB and skeleton-based features, the system achieves robust activity recognition. Experimental results demonstrate the model’s effectiveness in distinguishing a wide variety of human activities with minimal misclassifications. The two-stream architecture excels in capturing intricate human interactions, particularly in scenarios involving motion variation and diverse viewpoints. Additionally, the model’s stability across training epochs underscores its strong generalisation capability. Despite promising results, challenges remain in distinguishing actions with similar motion characteristics, such as “Punch” and “Hug”. Future work will explore advanced feature extraction techniques, such as attention mechanisms or graph-based models, to improve the model’s ability to capture subtle differences between actions. We also plan to evaluate the system in real-time applications and explore additional modalities, such as depth information, to enhance robustness in dynamic environments.

## Figures and Tables

**Table 1:** Augmentation techniques and possible ranges.

Augmentation Technique	Range
Rotation range	20
Width shift range	0.2
Height shift range	0.2
Shear range	0.2
Zoom range	0.2
Horizontal flip	True
Fill mode	Nearest

**Table 2:** Model architecture and its parameters.

Layer (Type)	Output Shape	Param	Connected to
lstm_input (InputLayer)	(None, 20, 102)	0	[]
input_1 (InputLayer)	(None, 20, 2048)	0	[]
lstm	(None, 20, 64)	42,752	[’lstm_input[0][0]’]
lstm_3	(None, 20, 64)	540,928	[’input_1[0][0]’]
lstm_1	(None, 20, 128)	98,816	[’lstm[0][0]’]
gru	(None, 8)	1776	[’lstm_3[0][0]’]
lstm_2	(None, 64)	49,408	[’lstm_1[0][0]’]
dropout	(None, 8)	0	[’gru[0][0]’]
dense	(None, 64)	4160	[’lstm_2[0][0]’]
dense_2	(None, 8)	72	[’dropout[0][0]’]
dense_1	(None, 32)	2080	[’dense[0][0]’]
concatenate	(None, 40)	0	[’dense_2[0][0]’,’dense_1[0][0]’]
dense_3	(None, 8)	328	[’concatenate[0][0]’]
Total params		Trainable params: 740,320	Non-trainable params: 740,320

**Table 3:** Ablation study of the proposed model with single- and multi-modality.

Dataset Name	Modality	Modality Name	Stream Name	Validation Accuracy	Validation Loss
TPIk Dataset	Single-Modality	Only Skeleton	Stream-1	71.00	1.82
TPIk Dataset	Single-Modality	Only RGB	Stream-2	95.00	0.14
TPIk Dataset	Multi-Modality	Skeleton+RGB	Stream-1 + Stream-2	96.56	0.14
HAR Video Dataset	Single-Modality	Only Skeleton	Stream-1	89.42	0.43
HAR Video Dataset	Single-Modality	Only RGB	Stream-2	94.00	0.14
HAR Video Dataset	Multi-Modality	Skeleton+RGB	Stream-1 + Stream-2	96.16	0.10

**Table 4:** Labelwise performance of the proposed model.

Task	Precision	Recall	F1 Score	Accuracy (%)
Close Up	98.36	98.36	98.36	98.36
Get Away	99.18	97.58	98.37	97.58
Kick	99.19	96.09	97.62	96.09
Push	99.07	99.07	99.07	99.07
Shake Hands	96.15	98.04	97.09	98.04
Hug	97.87	90.20	93.88	90.20
Give a Notebook	91.74	97.37	94.47	97.37
Punch	89.77	91.86	90.80	91.86
Average	96.42	96.07	96.21	96.56

**Table 5:** State of the art comparison of the proposed model.

Author	Dataset Name	Feature Extraction	Classifier	Accuracy (%)
Yun et al. [10]	Two Person Interaction Kinect Dataset	Joint feature	SVM	80.03
Yun et al. [10]	Two Person Interaction Kinect Dataset	Plane feature	SVM	73.80
Yun et al. [10]	Two Person Interaction Kinect Dataset	Velocity feature	SVM	48.03
Yun et al. [10]	Two Person Interaction Kinect Dataset	Joint+plane feature	SVM	79.00
Yun et al. [10]	Two Person Interaction Kinect Dataset	Joint+velocity	SVM	80.02
Yun et al. [10]	Two Person Interaction Kinect Dataset	Velocity+plane	SVM	74.44
Yun et al. [10]	Two Person Interaction Kinect Dataset	All feature	SVM	79.00
Hu et al. [11]	Two Person Interaction Kinect Dataset	Joint, plane, velocity	SVM	81.67
Hu et al. [11]	Two Person Interaction Kinect Dataset	Joint, plane, velocity	MIL	83.33
Saha et al. [12]	Two Person Interaction Kinect Dataset	Rotation invariance Rotation variance phenomenon	SVM	90.00
Li et al. [16]	Two Person Interaction Kinect Dataset	Torso (forward, back, still, turning), Hand (left right), Arm (right, left with free and raised), Leg(forward, back, kick, still, both)	LSTM	90.40
Li et al. [16]	Two Person Interaction Kinect Dataset	Torso (forward, back, still, turning), Hand (left right), Arm (right, left with free and raised), Leg(forward, back, kick, still, both)	LSTM-CRF	90.60
Proposed Method	Two Person Interaction Kinect Dataset	Two-stream DL	DL	96.56

**Table 6:** Labelwise performance of the proposed model for the HAR Video Dataset.

Task	Precision (%)	Recall (%)	F1 Score (%)	Accuracy (%)
Clapping	100.00	100.00	100.00	100.00
Meet and Split	100.00	100.00	100.00	100.00
Sitting	100.00	100.00	100.00	100.00
Standing Still	100.00	100.00	100.00	100.00
Walking	91.67	94.29	92.96	94.29
Walking While Reading Book	100.00	94.44	97.14	94.44
Walking While Using Phone	86.67	89.66	88.14	89.66
Combined Features	96.90	96.91	96.89	96.16

## Data Availability

Two Person Interaction Kinect Dataset: (https://www.kaggle.com/datasets/dasmehdixtr/two-person-interaction-kinect-dataset (accessed on 31 October 2024), Human activity recognition (HAR) video dataset, sourced from Kaggle (available at https://www.kaggle.com/datasets/sharjeelmazhar/human-activity-recognition-video-dataset (accessed on 31 October 2024).

## Abbreviations

LSTM	Long short-term memory
BiLSTM	Bi-directional long short-term memory
CNN	Convolutional neural networks
GMM	Gaussian mixture model
DD-Net	Double-feature double-motion network
GAN	Generative adversarial network
SOTA	State of the art
MobileNetV2	Mobile network variant 2
ReLU	Rectified linear unit
DCNN	Dilated convolutional neural network
GRNN	General regression neural network
KFDI	Key frames dynamic image
QST	Quaternion spatial-temporal
RNNs	Recurrent neural networks
BT-LSTM	Block-term long-short memory
KF	Kalman filter
KM-Model	Keypoint mesh model
SRGB-Model	Segmented RGB model
